# E-cigarette use and intentions related to psychological distress among cigarette, e-cigarette, and cannabis vape users during the start of the COVID-19 pandemic

**DOI:** 10.1186/s40359-022-00910-9

**Published:** 2022-08-15

**Authors:** Patricia Cabral

**Affiliations:** Department of Psychology, Occidental College, 1600 Campus Road, Los Angeles, CA 90041 USA

**Keywords:** Smoking, E-cigarette use, Cannabis use, Psychological distress, Intentions to use e-cigarettes

## Abstract

**Background:**

This study examines associations between psychological distress, intentions to use e-cigarettes, and cigarette smoking, e-cigarette use, and cannabis consumption through e-cigarette use among a diverse sample of U.S. young adults.

**Procedures:**

Young adults (*N* = 314; 72.5% female) were recruited to complete an online survey during the first few months of the COVID-19 pandemic.

**Results:**

Associations between psychological distress and cigarette smoking, e-cigarette use, cannabis vaping, and intentions to use e-cigarettes were found. Current e-cigarette use (OR = 1.23, 95% CI 1.17, 1.28, *p* < .001; 7.5%) and cannabis vaping (OR = 2.03, 95% CI 1.88, 2.18, *p* < .001; 10%) was higher among female, possibly due to the significantly higher psychological distress reported among females. Interactions between intentions to use e-cigarettes and psychological distress variables were found for all smoking and vaping behaviors.

**Conclusions:**

Public health efforts should increase focus on providing psychological services for young adults to improve coping strategies that are alternative to smoking and vaping behaviors.

## Introduction

E-cigarette use has become a major health concern among young adults. In 2018, the estimated prevalence of young adult (ages 18–24) current electronic cigarette (e-cigarette) users was 7.6% [1]. More than half of e-cigarette users are under the age of 35 [2]. Moreover, the COVID-19 pandemic has placed additional health burdens among cigarette smokers and e-cigarette users. In fact, smokers and e-cigarette users are significantly more likely to report COVID-19 symptoms [3–5]. Moreover, the predominant use of—and preference for—e-cigarette products among young adults presents an additional concern due to increased psychological distress during the COVID-19 pandemic [6]. Increased rates of psychological distress during the early COVID-19 pandemic may have facilitated smoking and e-cigarette use as well as intentions to use e-cigarettes. Thus, an examination of the role of psychological distress and intentions to use e-cigarettes as an underlying mechanism of smoking and e-cigarette use among young adults in the U.S. during the early pandemic is needed.

### Increased risk of COVID-19 outcomes for smokers and e-cigarette users

Evidence suggests that current cigarette smokers are at an increased risk of COVID-19 outcomes due to enhanced expression of angiotensin-converting enzyme-2 (ACE-2), which is the gene encoding the receptor for severe acute respiratory syndrome coronavirus 2 (SARS-CoV-2), throughout the human airway tract [7]. Not only are cigarette smokers more likely to report symptoms of COVID-19, but they are also more likely to be hospitalized if they test positive for COVID-19 [4, 5]. For example, heavy smokers (i.e., more than 30 pack-years) have higher odds of hospitalization and death following a COVID-19 diagnosis when compared to non-smokers [8]. Additionally, men who smoke have a higher expression of ACE-2 than women, possibly explaining higher COVID-19 mortality rates among males [9, 10]. Sex differences in ACE-2 expression have also been found for e-cigarettes use [11].

Although not yet extensively studied as cigarette smoking exposure, studies examining e-cigarette use have begun to demonstrate a similar association with COVID-19 risk [12]. Often referred to as “vaping” because many people incorrectly believe e-cigarettes create a vapor that is then inhaled but, in fact, produce an aerosol of tiny particles, e-cigarettes have become a growing public health concern among adolescents and young adults [13–15]. Specifically, a greater preference for e-cigarettes among youth [2, 16], which are often perceived to be safer than traditional cigarette products [17, 18], highlights a need for empirical work to explore the use of vaping products during a highly distressing time (i.e., the early COVID-19 pandemic months). In fact, e-cigarette use, or “vaping”, seems to be associated with COVID-19 diagnosis among adolescents and young adults [3].

E-cigarettes, or vaping products, may be used in a variety of methods and for various purposes. For example, some e-cigarette users will concurrently smoke cigarettes or use vaping to consume cannabis products. In fact, a large proportion of e-cigarette users report a preference for “vaping” cannabis as opposed to smoking it [19], particularly among young adults [20]. When examining the risk of COVID-19 diagnosis among cigarette smokers, e-cigarette users, and dual users (i.e., individuals who use both cigarettes and e-cigarettes), COVID-19 diagnosis was five times more likely among exclusive e-cigarettes users and seven times more likely among dual-users [3]. Moreover, some studies have suggested that concurrent use of e-cigarettes and cannabis may be an underlying risk factor of COVID-19 symptomatology and diagnosis, in that, compared to exclusive e-cigarette users, concurrent e-cigarette and cannabis users were more than three times more likely to report COVID-19 symptoms and almost twice as likely to report a COVID-19 diagnosis [21]. Taken together, growing evidence shows an increased risk of COVID-19 outcomes due to cigarette smoke and e-cigarette “vaping”. Furthermore, these associations emphasize a need to illuminate the role of psychological distress in driving the use of cigarette and vaping products during the early COVID-19 pandemic.

### Intentions to use e-cigarettes

According to the Theory of Planned Behavior [22, 23], a person’s intentions to engage in a behavior are a major determinant in behavioral engagement. It is well established that intentions to engage in behaviors are important predictors of future smoking behaviors among youth [24, 25], such that intentions to use e-cigarettes are related to current e-cigarette and cigarette use [26]. Moreover, e-cigarette use appears to increase the likelihood of cigarette smoking among youth [27], by way of increasing intentions to smoke cigarettes when youth have used e-cigarettes [28, 29]. Additionally, intentions to quit using e-cigarettes are lower among youth dual users as opposed to exclusive e-cigarette users [30]. Thus, intentions to use e-cigarettes are important determinants of future behaviors which may increase the likelihood of e-cigarette use as well as cigarette smoking behaviors.

Furthermore, the increased risk that COVID-19 poses to smokers, and the increased experiences of psychological distress during the pandemic, may influence behavioral intentions. For example, among a sample of Italian adults during the COVID-19 lockdown in April 2020, most exclusive cigarette smokers reported considering quitting, but most exclusive e-cigarette users had not considered stopping the use of e-cigarettes [31]. Thoughts of quitting appear to differ according to smoking or vaping method and may closely reflect intentions. Indeed, past work has found that more than 10% of youth had intentions to use e-cigarettes and smoke cigarettes [32, 33]. Alternatively, more than half of current e-cigarette young adult users want to quit [34]. Yet, many cigarettes and e-cigarettes users may find it difficult to quit because psychological distress can make it more difficult to succeed [35].

### Psychological distress related to smoking and vaping behaviors

The pandemic has led to significant increase in psychological distress (i.e., anxiety, depression, stress) [36]. Among the general U.S. adult population during the spring of 2020, clinically significant levels of anxiety and depression were reported, particularly among young adults [37–39]. Persistently elevated psychological distress among U.S. adults spanning from April 2020 through August 2021 in comparison to consistent prevalence rates of 3–4% prior to the onset of the COVID-19 pandemic demonstrate the ongoing impact on mental health [40]. Although several studies have reported a decline or cessation of e-cigarette use among many populations during the pandemic because of factors such as lack of accessibility due to stay-at-home orders and smoke shop closures, some proportion of young adults increased their use of e-cigarettes [41, 42]. For example, since the start of the pandemic, 24% of smokers increased their cigarette use and 28% decreased [43]. Conversely, over 27% of e-cigarette users had increased their e-cigarette use and less than 24% had decreased [43]. Differences in reduction rates between cigarette and e-cigarette use may reflect underlying mechanisms such as age, risk perceptions [44], nicotine dependence [45], and a possible preference for e-cigarette use as a coping strategy for psychological distress among youth. Yet, most studies have not examined the role of psychological distress as a facilitating factor in the use of e-cigarette use among current young adult users since the start of the pandemic.

As smoking behaviors are often precipitated by reports of high anxiety and stress [46–48], it may also lead to an increase in smoking and the use of e-cigarettes among young adults during the pandemic. In fact, college students often report using e-cigarettes as a stress management tool [49]. Moreover, among youth, loneliness has been positively associated with smoking [50] and e-cigarette use [51]. Similarly, depression is associated with higher smoking dependence [52] and appears to account for difficulty in smoking cessation efforts as rates remain consistently lower for depressed smokers than for smokers in the general population [53]. However, there is a lack of research examining whether increases in psychological distress of anxiety, depression, loneliness, and stress, during the early months of the COVID-19 pandemic influenced smoking, e-cigarette use, and intentions to use e-cigarettes simultaneously among young adults in the U.S.

### Current study

The current study aims to examine rates of cigarette smoking, e-cigarette use, dual usage, and cannabis vaping during the early months of the COVID-19 pandemic, well as the influence of intentions to use e-cigarettes and psychological distress (i.e., anxiety, depression, loneliness, and stress) on smoking and vaping behaviors among young adults. Due to the increase in popularity of electronic devices for smoking [2], this study primarily focused on the intentions and use of e-cigarettes. However, cigarette smoking was also assessed as an outcome. Moreover, due to marked differences in smoking behaviors between men and women [54], associations were examined separately for gender. Four hypotheses were proposed: (H1) Current cigarette smoking prevalence will be lower than e-cigarette use rates among young adults, but both rates will be higher among men than women. (H2) Intentions to use e-cigarettes in the future will be higher among dual cigarette and e-cigarette users as well as those who use e-cigarettes to vape cannabis in comparison to current exclusive cigarette or exclusive e-cigarette users. (H3) Moreover, intentions to use e-cigarettes will be positively associated with cigarette smoking, e-cigarette use, and the use of e-cigarettes to consume cannabis among both men and women. Last, (H4) psychological distress, including anxiety, depression, loneliness, and stress will be positively associated with current cigarette smoking, e-cigarette use, dual use, and consumption of cannabis through e-cigarettes by interacting with intentions to use e-cigarettes.

## Methods

### Participants

Participants for this study were recruited between September 2019 to December 2020 and included 405 emerging adults (M age = 20.35; SD = 2.71) from the greater Los Angeles area. Analyses for this study included respondents who participated from March 23, 2020 to November 25, 2020 (*N* = 314; *M* age = 20.56, SD = 2.97; 73% females), after the World Health Organization (WHO) declared COVID-19 a global pandemic on March 11, 2020. Respondents collected prior to the pandemic from September 14, 2019 to December 2, 2019 (*n* = 91) were excluded from analyses as this study focused on processes during the pandemic. Seventy-two percent of the sample were current college students. The sample was diverse in racial/ethnic background (22% Asian; 4.2% Black; 11.9% Latina/o; 1.5% Pacific Islander; 1.7% Other; 45.7% White; 13% Multiracial), but predominantly identified as female (72.5%). Most participants identified as Heterosexual (70%), followed by Bisexual (17%), Gay/Lesbian (7%), Queer (1.7%), and Other/Prefer not to say (4.3%). The median household income was between 80,000 to 89,999 per year. Over 30% of participants reported their parents’ (both mother and father) education attainment to be a 4-year college degree.

### Measures

Item questions and statements for variables measured can be found in Table 1.

**Table 1:** Item questions and statements for variables measured

Variable	Item question/statement
Cigarette smoking	Do you currently smoke tobacco products such as cigarettes?
E-cigarette use	“Do you know of electronic cigarettes?”
Current E-cigarette use	“Are you currently using electronic cigarettes?”
Recent cannabis consumption	“During the last 30 days, on how many days did you take cannabis? (___days/30)”
Cannabis vaping	“Which of the following substances did you inhale with your electronic cigarette?”
E-Cigarette intentions	“In the future, do you intend to use the electronic cigarette?”
Intentions for continued use of E-cigarettes	“For how much longer do you intend to use the electronic cigarette?”
Anxiety	“During the past month, how often have you felt anxious?”
Depression	“During the past month, how often have you felt depressed?”
Loneliness	“During the past month, how often have you felt lonely?”
Stress	“During the past month, how often have you felt stressed?”
E-cigarette use to relieve anxiety	“Does the e-cig relieve anxiety, nervousness?”
E-cigarette use to relive stress	“I use[d] the electronic cigarette… – to deal with stress”

#### Cigarette smoking and e-cigarette use

Current cigarette smoking was assessed using a single item (see Table 1 for item questions and statements) responses on a 4-point scale (1 = “No, I never was a smoker”; 2 = “No, I am an ex-smoker”; 3 = “Yes, occasionally but not every day”; 4 = “Yes, I smoke every day”). Participants who reported occasional and frequent current use of cigarettes were combined and dichotomized (0 = “No”, 1 = “Yes”) to estimate the prevalence of overall current cigarette smokers across usage levels. Prevalence of ever having used an e-cigarette was measured using a single question regarding knowledge of e-cigarettes responses (1 = “No, I have never read anything about it and nobody told me about it”; 2 = “Yes, I read a bit about it or someone told me about it”; 3 = “Yes, I am informed on the e-cigarette, but I have never used it”; 4 = “Yes, and I have already used an e-cigarette”). Participants who responded “Yes, and I have already used an e-cigarette” were used to calculate the prevalence of ever e-cigarette use. Current e-cigarette use was assessed using a single item responses on a 5-point scale (1 = “No, I have never used it”; 2 = “No, but I have used it in the past”; 3 = “Yes, occasionally”; 4 = “Yes, I use it frequently but not daily”; 5 = “Yes, I use it every day”). Occasional, frequent, and daily current use of e-cigarettes were combined and dichotomized (0 = “No”, 1 = “Yes”) to estimate the prevalence of overall current users of e-cigarettes across usage levels.

Dual users of cigarettes and e-cigarettes were identified by coding (1 = exclusive cigarette user; 2 = exclusive e-cigarette user; 3 = dual user) based on responses to both items of current e-cigarette use and current cigarette smoking who responded with any of the affirmative answers to both questions (i.e., “Yes, occasionally”; “Yes, I use it frequently but not daily”; “Yes, I use it every day”).

#### Cannabis consumption

Cannabis consumption and use of e-cigarettes to consume cannabis products were each assessed using single items. Recent cannabis consumption was measured using the frequency of cannabis consumption in the last month, in which participants entered a numerical response to indicate the number of days cannabis was consumed in the last 30 days. Responses were dichotomized to indicate whether the participant was a current cannabis user (0 = “no use in the past 30 days”, 1 = “at least one day of use in the last 30 days”). The use of e-cigarettes to consume cannabis was measured as a discrete response to a single item question. Participants chose from various substances in their response including cannabis, cocaine, heroin, amphetamines, ecstasy, benzodiazepines/barbiturates, nicotine, and other. Participants who selected cannabis, regardless of their use of additional non-cannabis substances, were coded to indicate use of e-cigarettes to consume cannabis (0 = non-cannabis use of e-cigarettes; 1 = cannabis use of e-cigarettes).

#### E-cigarette intentions

Participants’ intentions to use e-cigarettes in the future, regardless of current and past e-cigarette use, were assessed using a single item on a 4-point response scale (1 = “I have no intention at all to use an e-cigarette”; 2 = “I don’t have plans to use or abstain from using an e-cigarette”; 3 = “I plan to use an e-cigarette”; 4 = “I strongly want to use an electronic cigarette”). Among current and past e-cigarette users, intentions to continue use of e-cigarettes was assessed using an additional single item about the length of time they intended to continue using e-cigarettes. Responses were on a 11-point scale (1 = “0 days”; 2 = “1 to 6 days”; 3 = “1 week”; 4 = “2 weeks”; 5 = “3 weeks”; 6 = “4 weeks”; 7 = “2 months”; 8 = “3 months”; 9 = “6 months”; 10 = “1 year”; 11 = “More than 1 year”).

#### Psychological distress

Anxiety, depression, loneliness, and stress were separately assessed as single item measures of psychological distress. Previous work has supported the use of single item measures of self-rated mental health to be highly associated with multi-item measures of health [55]. Perceived anxiety, depression, loneliness, and stress were assessed using single items of each variable as reported for symptoms in the past month. Responses for all psychological distress measures were on a 5-point scale (1 = “Never”; 2 = “Rarely”; 3 = “Sometimes”; 4 = “Often”; 5 = “Always”). Additionally, two items were used to measure the use of e-cigarettes to relieve anxiety and stress. Responses for the use of e-cigarettes to relieve anxiety were on a 6-point scale (1 = “Not applicable, I don’t experience nervousness, anxiety”; 2 = “No”; 3 = “Maybe”; 4 = “Yes, somewhat”; 5 = “Yes, a lot”; 6 = “Yes, definitely”) and responses for the use of e-cigarettes to relieve stress were on a 4-point scale of agreement (1 = “Not at all true”; 2 = “Not very true”; 3 = “Somewhat true”; 4 = “Very true”).

### Procedure

Participants completed an online survey through Qualtrics. The survey included questions on psychosocial determinants (e.g., attitudes, beliefs, other non-tobacco substance use, family history of smoking, peer influence, cultural influence, psychological distress, use to relieve anxiety and stress, weight and stress control), structural factors (e.g., age of initiation, attempts to quit, cost, modification of e-cigarette, flavor preferences, “vaping tricks”, social media vaping viewership, accessibility), and outcomes (i.e., oral and respiratory health such as coughing, throat irritation) of cigarette and e-cigarette use. Informed consent from all participants and IRB approval for the study was obtained through the Human Subjects Committee at Occidental College prior to data collection.

Participant recruitment for the survey was conducted across the 2019–2020 academic year and was open to all young adults over the age of 18. Fall 2019 data collection was completed by December 3 2019, prior to the World Health Organization (WHO) initial report of COVID-19 on December 31, 2019. Spring 2020 data collection began March 23, 2020 after the WHO declared COVID-19 a global pandemic on March 11, 2020. Thus, time of survey completion was used to determine inclusion for analyses (i.e., data collected prior to the WHO declaring COVID-19 a pandemic were excluded), with Spring survey responses used for data analyses.

### Statistical analysis

Prevalence of cigarette and e-cigarette use as well as psychological distress differences between gender were examined using chi-square and independent samples *t* tests, respectively. Differences in psychological distress and intentions to use e-cigarettes across exclusive cigarette (70% female; *M* age = 20.89, SD = 1.62), exclusive e-cigarette users (85.7% female; *M* age = 21.50, SD = 4.88), dual users (56.3% female; *M* age = 21.81, SD = 8.42), and cannabis vapers (76.7% female; *M* age = 20.53, SD = 2.06) were examined using ANCOVAs. Logistic regressions were used to examine associations between major variables of interests with dichotomous outcomes, represented as odds ratios. Linear regressions were used for continuous outcomes, represented as beta coefficients. Age, race/ethnicity, household income, and parental (mother and father) educational attainment levels were controlled for in all regression analyses. Analyses that examined associations among the total sample additionally controlled for gender. All analyses were performed using weights to adjust for differential representation in the gender population in the sample.

## Results

Prevalence and descriptive data for all major variables of interest are provided in Table 2. Gender differences for major variables can be seen in Table 3.

**Table 2:** Prevalence and descriptive statistics of cigarette, e-cigarette use, intentions to use e-cigarettes, cannabis consumption, and psychological distress

	Overall (n = 314)	Female (n = 214)	Male (n = 100)
*Demographics*			
Age, M (SD)	20.49 (2.51)	20.61 (2.74)	20.13 (1.52)
Household income, Median	100–150 k/year	100–150 k/year	100–150 k/year
*Cigarette use*			
*Current use, %	11.0	9.4	16.0
Never, %	75.8	81.6	70.7
Ex-smoker, %	9.6	9.0	13.3
Occasional smoker, %	8.3	7.2	12.0
Daily user, %	2.5	2.2	4.0
*E-cigarette use*			
Ever use, %	39.2	39.5	42.7
*Current use, %	11.0	7.5	4.5
Never	55.7	59.2	56
Past user	29.6	30.0	32.0
Occasional user	2.9	1.8	6.7
Frequent user	5.1	3.6	1.3
Daily user	5.3	5.4	4.0
^a^Used to relieve anxiety, M (SD)	3.46 (1.4)	3.33 (1.44)	4.10 (1.13)
^b^Used to deal with stress, M (SD)	2.08 (1.09)	2.18 (1.14)	1.74 (0.89)
*Intentions to use e-cigarettes*			
Intentions, %			
No intentions to use	77.6	78.4	74.8
No plans to use or abstain	18.2	16.8	22.5
Plan to use e-cigarette	3.4	3.7	2.3
Strongly want to use e-cigarette	0.9	1.0	0.4
^c^Intended length of time for future use, M (SD)	5.19 (3.51)	5.13 (3.50)	6.0 (3.83)
*Exclusive versus dual use*			
Exclusive cigarette user, %	5.2	4.8	6.3
Exclusive e-cig user, %	5.2	6.1	2.5
Dual user, %	5.5	4.4	8.8
*Cannabis*			
Past 30 Days Use, M number of days (SD)	9.47 (10.71)	9.12 (10.65)	10.48 (10.94)
Cannabis vaping, %	9.4	10	7.5
^d^*Psychological distress, M (SD)*			
Anxiety	3.31 (1.09)	3.40 (1.08)	3.04 (1.07)
Depression	2.93 (0.96)	2.99 (0.99)	2.76 (0.84)
Loneliness	2.93 (1.02)	2.98 (1.04)	2.08 (0.93)
Stress	3.65 (0.88)	3.72 (0.85)	3.45 (0.92)

Prevalence reported represent weighted percentages

*Current use represents the combined prevalence of occasional, frequent, and everyday users

^a^Response range: 1 = Not applicable, I don’t experience nervousness, anxiety; 2 = No; 3 = Maybe; 4 = Yes, somewhat; 5 = Yes, a lot; 6 = Yes, definitely

^b^Resonse range: 1 = Not at all true; 2 = Not very true; 3 = Somewhat true; 4 = Very true

^c^Response range: 1 = 0 days, 2 = 1 to 6 days, 3 = 1 week, 4 = 2 weeks, 5 = 3 weeks, 6 = 4 weeks, 7 = 2 months, 8 = 3 months, 9 = 6 months, 10 = 1 year, 11 = More than 1 year

^d^Response range: 1 = Never; 2 = Rarely; 3 = Sometimes; 4 = Often; 5 = Always

**Table 3:** Independent samples *t* test examining differences between gender for all major variables

	Females		Males		*T* test
	M	SD	M	SD	
Ever used cigarettes	0.11	0.31	0.18	0.38	21.16***
Current cigarette use	0.01	0.11	0.02	0.15	8.45***
Ever used e-cigarettes	0.37	0.48	0.37	0.48	1.65
Current e-cigarette use	0.07	0.26	0.04	0.20	− 15.45***
Dual cigarette & e-cigarette use	0.07	0.26	0.25	0.43	11.98***
Cannabis vaping	0.29	0.09	0.05	0.22	− 18.47***
Intentions to use e-cigarettes	1.27	0.58	1.28	0.52	2.04*
Intended length of time for future e-cigarette use	5.13	3.50	6.0	3.83	3.37***
Past 30-day cannabis use	8.33	10.13	10.26	10.69	15.14***
Intentions to use e-cigarettes among cannabis users	1.45	0.72	1.56	0.61	12.10***
Intended length of time for future e-cigarette use among cannabis users	2.63	2.91	1.46	1.98	− 20.23***
Anxiety	3.40	1.08	3.04	1.07	− 38.35***
Depression	2.99	0.99	2.76	0.84	− 30.58***
Loneliness	2.98	1.04	2.08	0.93	− 23.18***
Stress	3.72	0.85	3.45	0.92	− 35.88***
E-cigarette use to relieve anxiety	3.33	1.44	4.10	1.13	39.26***
E-cigarette use to relieve stress	2.18	1.14	1.74	0.89	− 29.51***

*p < .05; ***p < .001

### Smoking and e-cigarette use

Among the analytical sample of participants recruited after WHO declared COVID-19 a pandemic (*n* = 314), 20.4% (18.4% females, 29.3% males) reported being a current or past cigarette smoker, 11% reported being a current smoker (i.e., “occasionally” and “daily”), 39.2% (39.5% females, 42.7% males) reported having ever used an e-cigarette, 11% reported current use of e-cigarettes (i.e., “occasionally”, “frequently”, and “daily”), and 77.7% reported currently being an abstainer from all cigarette and e-cigarette behaviors. Chi-square analyses show that the prevalence of ever having used e-cigarettes was significantly higher than the prevalence of ever having smoked a cigarette (χ^2^ = 6074.0, *df* = 1, *p* < 0.001). A multinomial logistic regression was used to estimate the likelihood of exclusive cigarette, e-cigarette, and dual usage based on gender. Males were more likely to report current cigarette use (OR = 1.26, S.E. = 0.10, 95% CI = 1.03, 1.54), less likely to be current e-cigarette users (OR = 0.44, S.E. = 0.05, 95% CI = 0.40, 0.48), and more than twice as likely to be dual cigarette and e-cigarette users (OR = 2.27, S.E. = 0.09, 95% CI = 1.91, 2.70) than their female counterparts. See Table 2 for details of cigarette and e-cigarette prevalence among males and females and Table 3 for *t* test results of gender differences.

### Intentions to use e-cigarettes

Over 6% of participants, regardless of smoking and e-cigarette use experience, indicated that they planned or wanted to use an e-cigarette in the future (“I plan to use an e-cigarette” = 5.5%; “I strongly want to use an e-cigarette” = 1.6%), with females reporting significantly higher intentions (see Table 3). An ANCOVA among all current cigarette and/or e-cigarette users show that exclusive cigarette users’ intentions to use e-cigarettes (*M* = 1.75, SD = 0.58) were significantly lower than exclusive e-cigarette users’ (*M* = 2.38, SD = 0.62) and dual users’ intentions (*M* = 2.76, SD = 0.83), but dual users’ intentions to use e-cigarettes were not significantly different from exclusive e-cigarette users’ intentions (*F* (1, 3) = 10,300.45, *p* < 0.001).

The largest proportion of participants (16%) reported no intentions to use e-cigarettes (0 days), followed by 2 weeks and 1 year (8% each). Among all current cigarette and e-cigarette users, intended length of time for future e-cigarette use significantly differed between females (see Table 3), with males reporting longer timeline intentions to use e-cigarettes. ANCOVA results showed that intended length of time for future use of e-cigarettes was significantly different among exclusive cigarette smokers (*M* = 1.43, SD = 1.05), exclusive e-cigarette users (*M* = 5.44, SD = 3.50), and dual users (*M* = 6.11, SD = 3.08), with cigarette users having the shortest intended length of use between 0 days and less than 1 week (*F* (2, 5) = 325.76, *p* < 0.001).

Logistic regressions were used to test associations between intentions to use e-cigarettes and current cigarette and e-cigarette use among females and males. Females were more likely to be current cigarette smokers if they reported intentions to use e-cigarettes (OR = 1.26, S.E. = 0.11, 95% CI = 1.02, 1.55), but the intended length of time for future e-cigarette use did not predict current cigarette usage (OR = 0.99, S.E. = 0.03, 95% CI = 0.94, 1.06). Conversely, intentions to use e-cigarettes did not predict current e-cigarette use (OR = 0.91, S.E. = 0.06, 95% CI = 0.80, 1.03) among females, yet the intended length of time for future e-cigarettes use predicted a higher likelihood of being a current e-cigarette user (OR = 2.87, S.E. = 0.03, 95% CI = 2.70, 3.04). Both intentions to use e-cigarettes (OR = 0.02, S.E. = 0.60, 95% CI = 0.01, 0.04) and intended length of time for future e-cigarettes use (OR = 1.76, S.E. = 0.11, 95% CI = 1.43, 2.16) predicted dual usage of cigarette and e-cigarettes among females. Among males neither type of intentions for e-cigarette use predicted current cigarette use nor e-cigarette use (*p* > 0.90).

### Cannabis use

Over 41% of participants, regardless of smoking and e-cigarette use experience, reported using cannabis in the past month for an average of 9 days (*M* = 9.47; SD = 10.71) and over 9% reported using e-cigarettes to consume cannabis (see Table 2). Also, males reported a significantly greater number of days consuming cannabis than females (see Table 3). In addition to a higher likelihood of e-cigarette use among females (OR = 1.23, S.E. = 0.02, 95% CI = 1.17, 1.28), chi-square results indicated that a larger prevalence of females reported using e-cigarettes to consume cannabis (χ^2^ = 261.59, *df* = 1, *p* < 0.001) than males. Specifically, a logistic regression revealed that females (10%) were more than twice as likely to report using e-cigarettes to consume cannabis (OR = 2.03, S.E. = 0.04, 95% CI = 1.88, 2.18) than males (7.5%).

Over 7% of cannabis users (cannabis use in the last 30 days ≥ 1 day) reported intentions to use e-cigarettes (“I plan to use e-cigarettes” = 5.8%; “I strongly want to use e-cigarettes” = 1.9%) and 2% reported intentions for long-term future use of e-cigarettes (i.e., more than 1 year). Among the overall sample, recent cannabis users (i.e., at least 1 day of cannabis use in the last 30 days) reported significantly higher intentions to use e-cigarettes (*M* = 1.48, SD = 0.69; *t* = − 18, 65, *df* = 15,037.11, *p* < 0.001) and lower intentions for the length of time to use e-cigarettes in the future (*M* = 2.36, SD = 2.76; *t* = 8.51, *df* = 1286.07, *p* < 0.001) in comparison to participants who had not used cannabis recently or at all (*M* intentions to use e-cigarettes = 1.33, SD = 0.59; *M* intended length of time for future e-cigarette use = 3.35, SD = 3.81). Males who use cannabis reported significantly higher intentions to use e-cigarettes, but females reported longer intentions of future e-cigarette use than males (see Table 3). MANCOVA results show that participants who use e-cigarettes to consume cannabis significantly differed in their intentions to use e-cigarettes (*M* = 2.40, S.E. = 0.04; *F* (4, 13,408) = 1798.85, *p* < 0.001) and the intended length of time for future e-cigarette use (*M* = 2.82, S.E. = 0.05; *F* (4, 13,408) = 1266.98, *p* < 0.001) than those who did not use cigarettes or e-cigarettes (*MD* = 0.74, S.E. = 0.01), exclusively used cigarettes (*MD* = 0.35, S.E. = 0.06), exclusively use e-cigarettes (*MD* = − 0.25, S.E. = 0.02), and dual users (*MD* = − 0.63, S.E. = 0.04; see Table 2 for descriptive data of intentions variables across groups).

Among females, linear regressions revealed that intentions to use e-cigarettes (β = 0.44, S.E. = 0.19, *p* < 0.001) and intended length of time for future e-cigarette use (β = 0.19, S.E. = 0.05, *p* < 0.001) predicted recent cannabis use (i.e., the last 30 days). Logistic regression indicated that intentions to use e-cigarettes predicted using e-cigarettes to consume cannabis (OR = 2.09, S.E. = 0.04, 95% CI = 1.33, 1.44), but intended length of time for future e-cigarette use did not predict using e-cigarettes to consume cannabis (*p* = 0.23). Among males, linear regressions revealed that intentions to use e-cigarettes (β = 0.35, S.E. = 0.39, *p* < 0.001) and intended length of time for future e-cigarette use (β = − 0.50, S.E. = 0.09, *p* < 0.001) predicted recent cannabis use. Also, a logistic regression demonstrated that intentions to use e-cigarettes (OR = 5006.81, S.E. = 0.58, 95% CI = 1590.28, 15,763.30) and intended length of time for future e-cigarette use (OR = 0.20, S.E. = 0.15, 95% CI = 0.15, 0.27) predicted the use of e-cigarettes to consume cannabis.

### Psychological distress

Over 90% of the total sample reported experiencing some level (i.e., “rarely”, “sometimes”, “often”, or “always”) of one or more type of psychological distress (i.e., anxiety, depression, loneliness, or stress). The highest frequency reported was for stress with 30% of participants reporting “sometimes”, 44% of participants reporting “often” and 15% “always”. Independent samples *t* test (see Table 3) results demonstrate that females reported significantly higher levels of anxiety, depression, loneliness, and stress than males.

A paired samples *t* test was used to compare levels of psychological distress. Among the overall sample, frequency of reported anxiety (*M* = 3.31, SD = 1.08) was significantly higher than frequency of depression symptoms (*M* = 2.94, SD = 0.96) in the last 30 days (*t* = 111.56, *df* = 71,337, *p* < 0.001). Anxiety (*M* = 3.31, SD = 1.09) was significantly higher than loneliness (*M* = 2.93, SD = 1.02; *t *= 97.26, *df* = 71,605, *p* < 0.001). Stress (*M* = 3.65, SD = 0.88) was significantly higher than anxiety (*M* = 3.31, SD = 1.09; *t* = − 106.66, *df* = 71,873, *p* < 0.001), depression (*M* = 2.94, SD = 0.96; *t* = − 224.99, *df* = 71,337, *p* < 0.001), and loneliness (*M* = 2.93, SD = 1.01; *t* =—202.58, *df* = 71,605, *p* < 0.001). Depression (*M* = 2.94, SD = 0.96) and loneliness (*M* = 2.93, SD = 1.01) were not significantly different from one another (*t* = 1.36, *df* = 71,609, *p* = 0.17). An ANOVA was used to assess differences in psychological distress between cigarette smokers, e-cigarette users, dual users, and cannabis vapers to individuals who did not engage in any of the behaviors. Participants who did not engage in any of the smoking or vaping behaviors reported significantly lower anxiety (*M* = 3.29, SD = 1.08; *F*(1,71,873) = 447.74, *p* < 0.001), depression (*M* = 2.91, SD = 0.96; *F*(1,71,605) = 631.13, *p* < 0.001), loneliness (*M* = 2.91, SD = 1.00; *F* (1, 71,605) = 260.26, *p* < 0.001), and stress (*M* = 3.65, SD = 0.88; *F* (1, 71,873) = 40.67, *p* < 0.001) than cigarette smokers (*M* anxiety = 3.40, SD = 0.92; *M* depression = 3.10, SD = 0.70; *M* loneliness = 2.90, SD = 1.05; *M* stress = 3.70, SD = 0.78), e-cigarette users (*M* anxiety = 3.86, SD = 1.13; *M* depression = 3.43, SD = 0.82; *M* loneliness = 3.36, SD = 1.11; *M* stress = 3.86, SD = 0.83), cannabis vapers (*M* anxiety = 3.31, SD = 1.02; *M* depression = 3.07, SD = 0.79; *M* loneliness = 2.85, SD = 1.14; *M* stress = 3.64, SD = 0.94), and dual cigarette and e-cigarette users (*M* anxiety = 3.53, SD = 0.89; *M* depression = 3.20, SD = 0.75; *M* loneliness = 3.13, SD = 0.96; *M* stress = 3.53, SD = 0.72). Logistic regressions were used to examine whether anxiety, depression, loneliness, and stress predict current cigarette use, e-cigarette use, dual usage of cigarette and e-cigarettes, as well as e-cigarettes to consume cannabis. Among females, anxiety (OR = 1.22, S.E. = 0.06, 95% CI = 1.09, 1.36), depression (OR = 1.32, S.E. = 0.06, 95% CI = 1.16, 1.49), loneliness (OR = 1.22, S.E. = 0.05, 95% CI = 1.10, 1.36) and stress (OR = 0.61, S.E. = 0.06, 95% CI = 0.54, 0.69) were significant predictors of current cigarette smoking. Anxiety (OR = 1.52, S.E. = 0.02, 95% CI = 1.45, 1.59), depression (OR = 1.46, S.E. = 0.0395% CI = 1.38, 1.54), loneliness (B = 0.87, S.E. = 0.02, 95% CI = 0.83, 0.91), and stress (OR = 0.64, S.E. = 0.03, 95% CI = 0.61, 0.68) were predictors of e-cigarette use. None of the psychological distress variables significantly predicted dual usage of cigarettes and e-cigarettes among females (*p* > 0.05). Anxiety (OR = 1.05, S.E. = 0.02, 95% CI = 1.01, 1.09), depression (OR = 1.92, S.E. = 0.03, 95% CI = 1.83, 2.02), loneliness (OR = 0.68, S.E. = 0.02, 95% CI = 0.65, 0.71) and stress (OR = 0.66, S.E. = 0.02, 95% CI = 0.63, 0.69) were significant predictors of using e-cigarettes to consume cannabis.

Among males, anxiety (OR = 1.41, S.E. = 0.08, 95% CI = 1.21, 1.64), depression (OR = 1.34, S.E. = 0.08, 95% CI = 1.14, 1.57), loneliness (OR = 1.26, S.E. = 0.06, 95% CI = 1.11, 1.42) and stress (OR = 0.76, S.E. = 0.09, 95% CI = 0.65, 0.91) were significant predictors of current cigarette smoking. Depression (OR = 3.05, S.E. = 0.07, 95% CI = 2.67, 3.48), loneliness (OR = 2.49, S.E. = 0.07, 95% CI = 2.19, 2.83) and stress (OR = 1.27, S.E. = 0.08, 95% CI = 1.10, 1.47) were significant predictors of current e-cigarette use. None of the psychological distress variables predicted dual usage among males. Anxiety (OR = 0.45, S.E. = 0.05, 95% CI = 0.40, 0.49), loneliness (OR = 0.90, S.E. = 0.05, 95% CI = 0.82, 0.99) and stress (OR = 5.46, S.E. = 0.06, 95% CI = 4.82, 6.17) were significant predictors of using e-cigarettes to consume cannabis.

Independent samples *t* test used to examine gender differences in the use of e-cigarettes to relieve anxiety and stress (see Table 3) revealed that endorsement of using e-cigarettes to relieve anxiety was significantly higher among males than females. Conversely, endorsement of using e-cigarettes to deal with stress was higher among females than males.

### Psychological distress and intentions to use e-cigarettes

Linear regressions were used to test whether psychological distress predicted intentions to use e-cigarettes. Among females, anxiety (β = 0.07, S.E. = 0.01, *p* < 0.001), depression (β = 0.11, S.E. = 0.01, *p* < 0.001), loneliness (β = − 0.20, S.E. = 0.01, *p* < 0.001), and stress (β = 0.04, S.E. = 0.01, *p* < 0.001) predicted intentions to use e-cigarettes. Also, anxiety (β = − 0.10, S.E. = 0.03, *p* < 0.001), depression (β = 0.09, S.E. = 0.04, *p* < 0.001), loneliness (β = − 0.24, S.E. = 0.04, *p* < 0.001), and stress (β = − 0.10, S.E. = 0.04, *p* < 0.001) predicted intended length of time for future e-cigarette use. Among males, anxiety (β = − 0.17, S.E. = 0.01, *p* < 0.001), depression (β = 0.10, S.E. = 0.01, *p* < 0.001), loneliness (β = 0.04, S.E. = 0.01, *p* < 0.001), and stress (β = 0.18, S.E. = 0.01, *p* < 0.001) predicted intentions to use e-cigarettes. Anxiety (β = 0.64, S.E. = 0.10, *p* < 0.001), depression (β = − 0.46, S.E. = 0.08, *p* < 0.001), loneliness (β = 0.68, S.E. = 0.08, *p* < 0.001), and stress (β = − 0.74, S.E. = 0.08, *p* < 0.001) predicted intended length of time for e-cigarette use.

Subsequently, interactions between intentions to use e-cigarettes and psychological distress factors were used to predict smoking and vaping behaviors. Among females, interactions between intentions to use e-cigarettes and anxiety (OR = 1.08, S.E. = 0.04, 95% CI = 1.00, 1.16) significantly predicted current cigarette smoking. Interactions between intentions to use e-cigarettes and anxiety (OR = 1.30, S.E. = 0.01, 95% CI = 1.26, 1.33) and depression (OR = 1.12, S.E. = 0.01, 95% CI = 1.09, 1.15) significantly predicted e-cigarette use. Interactions between intentions to use e-cigarettes and anxiety (OR = 1.06, S.E. = 0.01, 95% CI = 1.04, 1.09) and depression (OR = 1.07, S.E. = 0.01, 95% CI = 1.04, 1.10) significantly predicted using e-cigarettes to consume cannabis. Interactions with length of time for future intentions to use e-cigarettes with anxiety (OR = 2.09, S.E. = 0.09, 95% CI = 1.74, 2.51), depression (OR = 0.83, S.E. = 0.03, 95% CI = 0.78, 0.87), and loneliness (OR = 0.79, S.E. = 0.05, 95% CI = 0.73, 0.87) significantly predicted cigarette use. Interactions with length of time for future intentions to use e-cigarettes with anxiety (OR = 0.65, S.E. = 0.04, 95% CI = 0.60, 0.70), depression (OR = 1.33, S.E. = 0.02, 95% CI = 1.28, 1.38), and loneliness (OR = 1.25, S.E. = 0.03, 95% CI = 1.18, 1.32) significantly predicted e-cigarette use. Interactions with length of time for future intentions to use e-cigarettes with anxiety (OR = 1.72, S.E. = 0.03, 95% CI = 1.61, 1.83) and loneliness (OR = 0.84, S.E. = 0.02, 95% CI = 0.81, 0.86) significantly predicted e-cigarettes to consume cannabis.

Among males, no psychological distress variables significantly interacted with intentions to use e-cigarettes to predict cigarette smoking. Interactions between intentions to use e-cigarettes and anxiety (OR = 1.45, S.E. = 0.17, 95% CI = 1.04, 2.03) and depression (OR = 0.77, S.E. = 0.11, 95% CI = 0.62, 0.96) significantly predicted current e-cigarette use. Anxiety (OR = 0.61, S.E. = 0.03, 95% CI = 0.57, 0.65), and depression (OR = 5.33, S.E. = 0.06, 95% CI = 4.74, 6.0) interactions with intentions to use e-cigarettes significantly predicted using e-cigarettes to consume cannabis. Interactions between intended length of time for future e-cigarette use and anxiety (OR = 0.14, S.E. = 0.18, 95% CI = 0.10, 0.19), depression (OR = 3.74, S.E. = 0.16, 95% CI = 2.74, 5.1), and loneliness (OR = 0.37, S.E. = 0.09, 95% CI = 0.31, 0.44) to predict current cigarette use. No interactions between psychological distress factors and intended length of time for future e-cigarette use predicted e-cigarette use. Depression (OR = 0.15, S.E. = 0.55, 95% CI = 0.05, 0.45) significantly interacted with intended length of time for future e-cigarette use in predicting e-cigarette use to consume cannabis.

## Discussion

The overarching aim of this paper was to illuminate the role of intentions and psychological distress (i.e., anxiety, depression, loneliness, and stress) on cigarette and e-cigarette use during the beginning of the COVID-19 pandemic. Specifically, this study examined associations between intentions to use e-cigarettes, psychological distress, and cigarette use, e-cigarette use, and the use of e-cigarettes to consume cannabis among young adults.

### **H1**

E-cigarette use will be higher than cigarette use, and both will be highest among men.

Results provide partial support for hypothesis 1. Prevalence of ever having smoked a cigarette was significantly lower than the prevalence of ever having used an e-cigarette among the overall sample. However, current use of cigarettes and e-cigarettes were equal in prevalence. Additionally, consistent with hypothesis 1, cigarette smoking and dual usage of e-cigarettes and cigarettes among males was significantly higher than females. Yet, contrary to previous reports of higher prevalence of e-cigarette use among male youth [56, 57], e-cigarette use was higher among females. Gender differences in e-cigarette use are often driven by various factors such as perceived stress, in that, the association of perceived stress with tobacco product use is higher among females than males [58]. The role of stress as an underlying mechanism of e-cigarette use among females is particularly relevant to this study as female participants reported significantly higher stress than males. Psychological distress differences, along with other factors (e.g., reinforcement of e-cigarette use among females through sensory appeal of flavor preferences [59]) may partially explain the higher prevalence of e-cigarette use among females during the early pandemic timeline.

### **H2**

Intentions to use e-cigarettes will be highest among dual users and cannabis vapers.

Results partially supported hypothesis 2. Although dual users did not significantly differ in their intentions to use e-cigarettes from exclusive e-cigarette users, both groups as well as cannabis vapers reported higher intentions to use e-cigarettes than exclusive cigarette users. Perhaps cigarette and e-cigarette users have established their preferred method of smoking or vaping with no inclination to switch methods. For example, some e-cigarette users have reported a difficult adjustment period when switching from smoking to vaping due to various barriers (i.e., lack of knowledge regarding the technical aspects of vaping products) [60], which may dissuade switching methods. More importantly, dual users of cigarettes and e-cigarettes report significantly longer-term intentions to use e-cigarettes in the future, suggesting they are the least likely to attempt to quit in the near future. This is a particular concern during the COVID-19 pandemic as dual users may be the most vulnerable to negative outcomes from COVID-19 exposure [3].

Although cannabis consumption facilitates e-cigarette use it does not appear to be the strongest correlate of e-cigarette use. This may be because cannabis vapers had higher intentions to use e-cigarettes than cigarette smokers and abstainers, but lower intentions than exclusive e-cigarette and dual users. Perhaps the various options available for cannabis consumption (i.e., inhalation, oral ingestion, topical) influence intentions of vaping cannabis among cannabis consumers and may reflect preferences in the method of cannabis consumption. In fact, cannabis delivery method preferences among young adults are wide ranging (e.g., hollowed-out cigars filled with marijuana known as a “blunt” [61], bongs, vaporizers, and edibles [62]). Despite not having the highest intentions to use e-cigarettes, cannabis users are still a major population of concern during the pandemic because cannabis use has been correlated with COVID-19 hospitalization due to shared genetic underpinnings [63].

### **H3**

E-cigarette use intentions will be associated with cigarette and vaping behaviors.

Results provide support for hypothesis 3. Intentions were significant predictors of cigarette, e-cigarette, and dual usage among females. Previous studies have found an association between e-cigarette use and cigarette smoking [27–29]. This study findings demonstrate this association through the underlying mechanism of intentions. Moreover, both intentions to use e-cigarettes and intended length of time for future e-cigarette use predicted dual usage of cigarette and e-cigarettes among females. Previous research has indicated that e-cigarette users who are non-cigarette smokers report high intentions to smoke cigarettes compared with their counterparts who had never used e-cigarettes [64], suggesting an association between e-cigarette use and cigarette smoking. Results from this study further illuminate the role of intentions, specific to e-cigarettes, which appear to influence cigarette smoking behaviors as well. Intentions to use e-cigarettes, as well as factors that encourage these intentions, may increase the risk of both e-cigarette use and cigarette smoking among young adults who are not current cigarette smokers. Although intentions did not significantly predict e-cigarette use among males, other facets of intentions not studied here may influence e-cigarette use among this population (e.g., intentions regarding the frequency or type of e-cigarette). It is important to understand how different facets of intentions to use e-cigarettes (i.e., intentions to use, length of time, frequency) predict e-cigarette and cigarette use as it may help identify youth who are the most vulnerable to vaping and smoking behaviors.

Furthermore, intentions to use e-cigarettes predicted cannabis vaping among females. Among males, intentions to use e-cigarettes and length of time for future intention to use e-cigarettes predicted cannabis vaping. These results clarify the gender differences in specific types of intentions that influence e-cigarette use for cannabis consumption. In line with previous research that shows females and males differ in their primary forms of cannabis use (e.g., edibles, blunts) [62], these results clarify the nuanced gender differences in factors that influence e-cigarette use for cannabis consumption. The link between cannabis use, vaping, and COVID-19 outcomes [21] may suggest a need for emphasis on cannabis vaping individuals and those with intentions to use e-cigarettes to be identified as high risk for negative COVID-19 outcomes.

### **H4**

Psychological distress will be positively associated with smoking, e-cigarette use, and cannabis vaping by interacting with intentions.

Rates of cigarette and e-cigarette use at the beginning of the pandemic appear to be, at least partially, influenced by psychological distress. Anxiety, depression, loneliness, and stress predicted intentions to use e-cigarettes as well as intended length of time for future e-cigarette use. Among other factors, psychological distress is positively associated with cigarette and e-cigarette use [65–68]. Findings from this study reveal that the influence of psychological distress applies to cannabis vaping as well as cigarette and e-cigarette use by way of intentions to use e-cigarettes. These findings may clarify reports that most young adults did not decrease their substance use (i.e., marijuana use) amid the pandemic [69], perhaps, in part, because of higher psychological distress. The association between psychological distress and e-cigarette use found in this study are further supported by the higher prevalence of anxiety, depression, loneliness, and stress reported among smokers and e-cigarette users than abstainers. Also, results revealed that females are more likely to report using e-cigarettes as well as using e-cigarettes to consume cannabis than their male counterparts. Gender differences in psychological distress may partially explain the higher likelihood of e-cigarette and cannabis vaping among females as they also reported significantly higher levels of psychological distress than males.

Previous work along with this study’s results suggest a bi-directional, and harmful, relationship between psychological distress and cannabis vaping. For example, results in this study reveal that depression is associated with e-cigarette use to consume cannabis. Moreover, past studies indicate that the use of cannabis, vaping, or other tetrahydrocannabinol (THC) products may carry unique risks for those “self-medicating” and make depression worse [70]. Thus, psychological distress may both facilitate cannabis vaping behaviors and exacerbate the use of e-cigarettes to consume cannabis. Furthermore, most young adult cannabis vapers did not change use during the early pandemic [71]. A lack of change in cannabis vaping may be due to psychological distress as suggested by this study’s results.

Finally, intentions related to e-cigarette use significantly interacted with psychological distress factors, and subsequently related to a higher likelihood of cigarette smoking, vaping and cannabis vaping. Intentions to use e-cigarettes appears to strengthen the association between psychological distress and smoking and vaping behaviors, particularly among females. In fact, cigarette smokers, e-cigarette users, dual users, and cannabis vapers reported higher anxiety, depression, stress, and loneliness than their abstaining counterparts. Smokers with psychological distress have previously been found to report higher intentions to switch to electronic cigarettes as a method to quit smoking [72]. The results of this study further clarify this relationship by demonstrating that psychological distress interacts with various types of intentions to use e-cigarettes which may not be directly related to smoking cessation. Moreover, psychological distress may increase the likelihood of vaping cannabis which may subsequently increase risk of negative COVID-19 outcomes. Also, the results of this study suggest that intentions to use e-cigarettes are related to a higher likelihood of cigarette smoking. Thus, psychological distress among non-current cigarette smokers may place them at higher risk to begin cigarette smoking if they have intentions to use vaping products. This is a particular concern as the pandemic’s impact continues and facilitates persistent elevated symptoms of psychological distress (e.g., depression [73]) which may continue to influence smoking and vaping behaviors.

The influence of psychological distress on smoking and vaping behaviors may not be unique to the pandemic. However, this study’s results show that this association is significantly present for young adults during the early months of the pandemic. Moreover, the influence of psychological distress on smoking and vaping behaviors is likely to interact with intentions to use e-cigarettes. Thus, individuals who have intentions to use e-cigarettes or to continue using e-cigarettes for a longer period of time if already using them are more likely to use e-cigarettes and possibly cigarettes as well when experiencing various types of psychological distress, including anxiety, depression, loneliness, and stress. In addition to typical negative outcome concerns of smoking and vaping behaviors (e.g., exposure to harmful carcinogens and other cancer inducing chemicals), the ongoing COVID-19 pandemic makes these individuals more vulnerable to negative health outcomes.

## Limitations

Although two types of intentions for e-cigarette use (i.e., intentions to use and length of time for future use) were examined, intentions for smoking cigarettes were not measured and, therefore, comparisons of intentions for cigarette and e-cigarette use could not be conducted. Additionally, psychological distress measures were single-item assessments which does not allow assessment of the severity. Also, psychological distress measures were not specific to the COVID-19 pandemic, nor did the study measure the use of e-cigarettes to relieve depression and loneliness. This limits the ability to assess whether psychological distress was directly related to concerns of the pandemic and whether e-cigarette use was intended for the relief of all psychological distress factors. It is unclear if documented increase in psychological distress [74–76] experienced by U.S. young adults is directly related to the pandemic and if there is an underlying mechanism of fear for COVID-19 risk among those who smoke cigarettes or use e-cigarettes.

## Future research

Future research should examine multiple types of intentions for substance use such as frequency and purpose (e.g., to quit other substance use behaviors or to self-medicate against psychological distress). Examining behavioral intentions as a multi-faceted factor of substance use can clarify the underlying mechanisms of smoking and vaping behaviors as well as better identify at-risk individuals. Also, future work should endeavor to examine whether intentions influence various forms of cannabis consumption that do not rely on e-cigarettes to clarify whether this association is based on cannabis use itself or the delivery method.

Smoking relapse rates among young adults and those who recently have attempted to abstain is typically high [77]. Reduced availability of cessation programming (e.g., access to helplines, cessation clinics, face-to-face support, and appointments with general practitioners) during the pandemic may have further hindered efforts and success at smoking cessation [78]. Future work should examine if increased psychological distress experienced during the pandemic has influenced relapse rates.

## Conclusions

Reducing smoking and vaping behaviors has become more important than ever since the pandemic began due to the increased risk of COVID-19 outcomes. According to results from this study, two factors that may provide guidance for better targeting interventions are psychological distress and intentions. In fact, a significant change in intentions can lead to behavioral change [79]. In addition, providing better mental health support among young adults may subsequently reduce cigarette and e-cigarette use. Yet, the pandemic led to reduced availability of resources for smoking cessation efforts [78] and healthier options for coping with psychological distress. Public health efforts that provide alternative positive coping strategies such as exercise and meditation may help decrease reliance on e-cigarettes for psychological distress management. Increased public resources for psychological services and smoking cessation should focus on young adult populations.

Finally, curbing excessive cannabis use may mitigate the impact of COVID-19. Intervention efforts aimed at reducing cannabis vaping behaviors may partially result in a reduction of cannabis use. However, because cannabis is often used to deal with psychological distress, reducing availability of cannabis may unintentionally result in other negative coping strategies such as cigarette smoking. Thus, efforts to reduce vaping and smoking behaviors must balance the need for interventions that help young people develop healthy and effective coping skills that do not unintentionally have negative impact on other psychosocial outcomes such as psychological distress.

## Data Availability

The datasets generated during the current study are not publicly available due to concerns of confidentiality concerns and the sensitive nature of information collected but are available from the corresponding author on reasonable request.
